# The Impact of Air Quality on Patient Mortality: A National Study

**DOI:** 10.3390/ijerph22071123

**Published:** 2025-07-16

**Authors:** Divya Periyakoil, Isabella Chu, Ndola Prata, Marie Diener-West

**Affiliations:** 1 Department of Epidemiology and Biostatistics, Bloomberg School of Public Health, Johns Hopkins University, Baltimore, MD 21205, USA; mdiener@jhu.edu; 2 Department of Electrical Engineering and Computer Science, University of California, Berkeley, CA 94720, USA; 3 Albert Einstein College of Medicine, Bronx, NY 10461, USA; 4 Center for Population Health Sciences, School of Medicine, Stanford University, Palo Alto, CA 94304, USA; itaylor@stanford.edu; 5 School of Public Health, University of California, Berkeley, CA 94720, USA; ndola@berkeley.edu

**Keywords:** air pollution, survival analysis, American Family Cohort, Cox Regression, AQI, primary care data, health informatics, big data, EHR, epidemiology, public health

## Abstract

**Introduction:** Air pollution is a risk factor for a variety of cardiopulmonary diseases and is a contributing factor to cancer, diabetes, and cognitive impairment. The impact on mortality is not clearly elucidated. **Objectives:** The goal of this study is to determine the impact (if any) of air pollution on the 5-year mortality of patients in the American Family Cohort (AFC) dataset. **Methods:** The AFC dataset is derived from the American Board of Family Medicine PRIME Registry electronic health record data. It includes longitudinal information from 6.6 million unique patients from an estimated 800 primary care practices across 47 states, with 40% coming from rural areas. The Environmental Protection Agency’s Air Quality Index (AQI) measures were downloaded for the study period (2016–2022). Using the Python library pandas, the AFC and EPA datasets were merged with respect to date, time, and location. Cox Regression Models were performed on the merged dataset to determine the impact (if any) of air quality on patients’ five-year survival. In the model, AQI was handled as a time-independent (time-fixed) covariate. **Results:** The group with AQI > 50 had an adjusted hazard of death that was 4.02 times higher than the hazard of death in the group with AQI ≤ 50 (95% CI: 3.36, 4.82, *p* < 0.05). The hazard of death was 6.73 times higher in persons older than 80 years of age (95% CI: 5.47, 8.28; *p* < 0.05) compared to those younger than 80 years of age. Black/African American patients had a 4.27 times higher hazard of death (95%CI: 3.47, 5.26; *p* < 0.05) compared to other races. We also found that regional effects played a role in survival. **Conclusions:** Poor air quality was associated with a higher hazard of mortality, and this phenomenon was particularly pronounced in Black/African American patients and patients older than 80 years of age. Air pollution is an important social determinant of health. Public health initiatives that improve air quality are necessary to improve health outcomes.

## 1. Introduction

Both short-term and long-term exposure to poor air quality adversely impact human health [1,2,3]. According to the World Health Organization (WHO), an estimated 2.4 billion people are subjected to dangerous levels of pollution daily. The combined effects of ambient air pollution and household air pollution are associated with 7 million premature deaths annually [4,5]. Air pollution is a risk factor for a variety of cardiopulmonary diseases and is a contributing factor to cancer, diabetes, and cognitive impairment [1,2,3].

A large case–control study utilizing Medicare data demonstrated that short-term exposure to PM 2.5 (particulate matter) and ozone was associated with higher mortality [6]. Another longitudinal study showed that long-term exposure to air pollutants was associated with increased mortality [7]. Additionally, the study found that persons of lower socioeconomic statuses are disproportionately burdened by the adverse impacts of air pollution [7].

Emerging evidence links chronic air pollution exposure to systemic inflammation, oxidative stress, and elevated risk for cardiopulmonary disease and cancer [8,9]. However, few studies have leveraged HER data to longitudinally assess the relationship between real-world air quality and mortality, particularly across diverse populations [10,11].

Although there have been significant steps forward to understand the effects of pollution on human and public health, several gaps remain. The effects of low levels of exposure to pollutants over a long period of time are not well understood. Additionally, the differential effects of pollution on vulnerable populations (older adults, persons of color, and others) that may make them even more susceptible to the negative impacts of pollution exposure have not been elucidated. More work in this area is essential to uncover which groups are at the highest risk of negative impacts due to pollution exposure and what puts them at high risk. Finally, while the global climate continues to change rapidly, little is known about the scope of interaction between climate and pollution exposure and how these factors may exert a combined effect on human health. While several large national health datasets exist and contain valuable patient information, most do not contain information about the air quality experienced by said patients. Similarly, government air quality datasets do not contain patient information. In this study, we sought to study the impact of air quality on 5-year mortality of patients by merging two massive datasets and analyzing the merged dataset to explore the underlying patterns. The Stanford University School of Medicine Institutional Board Review reviewed and approved this study.

## 2. Data and Methods

### 2.1. Data Sources

The American Family Cohort (AFC) dataset consists of electronic health record data (EHR) from the American Board of Family Medicine (ABFM) PRIME Registry [12]. ABFM PRIME is an “outpatient Qualified Clinical Data Registry certified by the Centers for Medicaid and Medicare Services (CMS) in 2016 to report clinical quality measures for federal and other reporting requirements” [12]. The AFC dataset was created as a collaborative effort between the ABFM and Stanford University as a “resource suitable for research, public health and science in the public interest” [12]. The AFC data, which include EHR data from over 6.6 million unique patients across the United States, have been cleaned, harmonized, and deidentified [12]. A key strength of the AFC is that it includes data on underserved populations, including persons from racial and ethnic minorities [12]. The dataset includes over 800 primary care practices across 47 states, with 40% of AFC patients residing in rural areas [12]. The current study utilizes the data from the AFC for the time period ranging from January 2016 through October 2022. This subset of the AFC data includes the entirety of the patient encounters during this time period, including visit notes, diagnoses (International Classification of Diseases) notes, insurance information, vital signs, and social history [12]. Another important strength of the AFC dataset is that it consists of a wide payer mix with patients on private insurance plans, Medicaid, and Medicare, thereby increasing the representation of vulnerable populations and the generalizability of the sample to the overall United States population.

The Environmental Protection Agency’s (EPA) Air Quality Index dataset is a publicly available dataset published on the EPA’s website [13]. The EPA’s Air Quality Index, or AQI, ranges from 0 to 500 [14]. An AQI of 0–50 is defined as “good”, 51–100 as “moderate”, 101–105 as “unhealthy for sensitive groups”, 151–200 as “unhealthy”, 201–300 as “very unhealthy”, and 301–500 as “hazardous” [14]. AQI is determined by five major pollutants as defined by the Clean Air Act. These pollutants are ground-level ozone, particulate matter (PM2.5 and PM10), carbon monoxide, sulfur dioxide, and nitrogen dioxide [14]. AQI is calculated by taking the maximum of the AQI measures calculated for these individual pollutants [14].

### 2.2. Data Preparation

Inclusion criteria: Only patients with at least five primary care visits during the period of interest (1 November 2016 to 16 October 2022) were included in the study. Patients remaining in the study for the full five years were assumed to be censored at five years. A total of 39,596 unique patients, with a total of 222,753 clinic visits during the study period, met the inclusion criteria. When the date of death was reported by the family, it was considered to be a Postmortem Documentation visit.

Dataset merging: All data pre-processing was performed with the programming language Python 3.11 by one of the authors (DKP), who is a software engineer trained in data science methods [15]. The data were merged using the Python library Pandas. By the end of this process, for every patient, there was an associated Air Quality Index (AQI) value corresponding to the patient’s location and date of entry into the study. The final merged dataset contained data about age, gender, race, year of entry into the study, region of the United States, air quality, and vital status for each of the 39,596 patients at their time of entry into the study.

### 2.3. Variables

#### 2.3.1. Exposure Data

The data analysis was restricted to the records associated with the first primary care visit during the study period for each unique patient. Each record consisted of the patient’s characteristics at the time of entering the study: age, gender, race, region, year of entry, AQI, and whether or not the patient was deceased. In this dataset, the covariates of interest were handled as time-independent (non-varying over time).

#### 2.3.2. Outcome Data

The data were set up as “time-to-event” data, where the event of interest was death. For patients who were administratively censored or lost to follow-up, time-to-event was defined as the time between study entry and the last primary care visit. For patients who died over the course of the study, time-to-event was defined as the time between study entry and death. Since we were studying 5-year survival, “deceased individuals” were defined as those who died prior to administrative censoring at 5 years post study entry. All other individuals were defined as “not deceased”. Any individual who was “not deceased” was censored at 5 years unless they were lost to follow-up before the end of the 5-year period post study entry; these individuals were censored at the last known time of follow-up.

### 2.4. Statistical Analysis

All statistical analysis was performed with the statistical software Stata 17.0 [16]. Age at study entry, race, gender, and AQI were binary variables. Age was dichotomized into two categories: younger than 80 years old (*n* = 36,809) and older than or equal to 80 years old (*n* = 2787). Gender was self-reported in the EHR as either female (*n* = 22,175) or male (*n* = 17,421). Similarly, race was dichotomized into two categories: Black/African American (*n* = 3019) and Not Black/African American (*n* = 36,577). AQI was dichotomized into two categories: AQI ≤ 50 (“Good”, according to the EPA) and AQI > 50. The distributions of age, gender, race, and AQI are presented in Table 1.

The region was handled as a disjoint categorical (nominal) variable. The region variable was divided into nine regions of the United States as defined by the United States Census Bureau [17]. The distribution of patients by region is presented in Table 1. Year of entry into the study, with values spanning from 2016 to 2022, was also handled as a disjoint categorical (nominal) variable.

Due to the COVID pandemic, there were very few study entrants from 2020 to 2022 compared to the other years, so 2020–2022 was combined into a single category. The distribution of patients by year of entry into the study is presented in Table 1.

To understand how the Air Quality Index would affect a patient’s five-year survival, a time-independent covariate analysis was conducted, in which air quality (AQI) during study entry was inputted as a covariate (and main exposure of interest) in a Cox Regression Model. Gender, race, age, location (region), and year of entry into the study were treated as time-independent variables. Model building was conducted through simple Cox models of each covariate; two variable models were then constructed, and confounding was assessed. Multivariable Cox Models were performed. Checks on the proportional hazards assumption were performed. The complementary log–log plots displayed roughly parallel curves across strata, suggesting that the proportional hazards assumption is reasonably met. Model selection was performed using the Akaike Information Criterion (AIC).

## 3. Results

### 3.1. Bivariate Analysis

**Figure 1:**
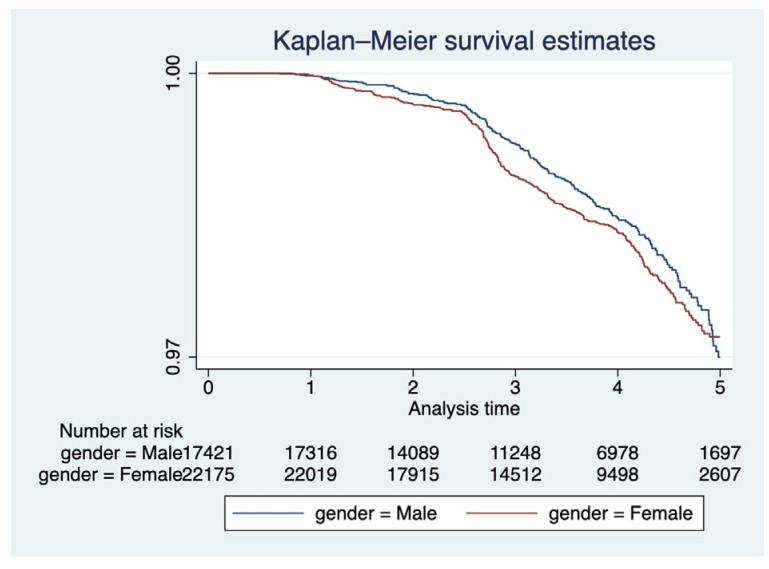
Kaplan–Meier survival curves by gender. Female participants demonstrated slightly higher survival over the follow-up period compared to males.

**Figure 2:**
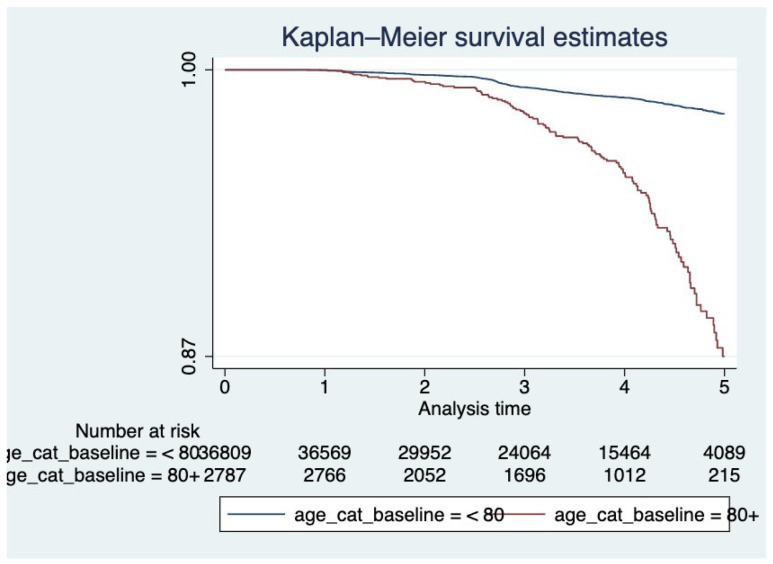
Kaplan–Meier survival curves by age group. Participants aged ≥80 at baseline had significantly lower survival over time compared to those aged <80.

**Figure 3:**
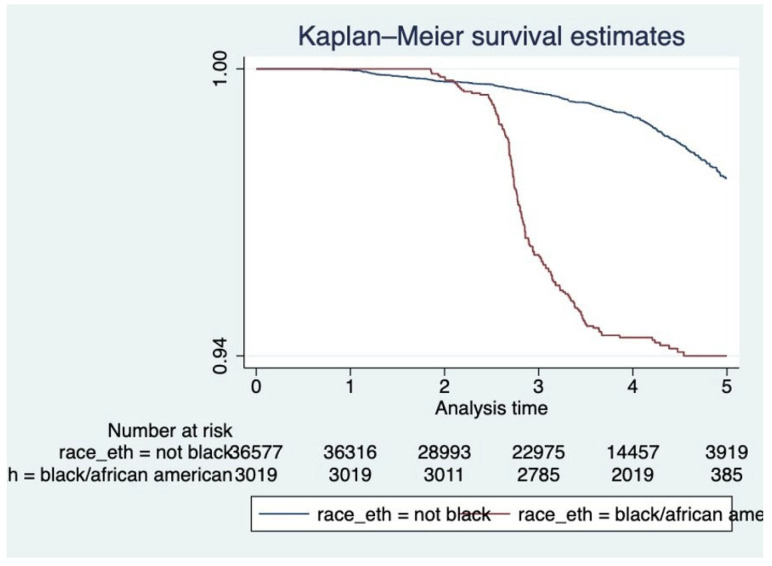
Kaplan–Meier survival curves by race/ethnicity. Black/African American participants showed markedly lower survival compared to non-Black participants over the follow-up period.

**Figure 4:**
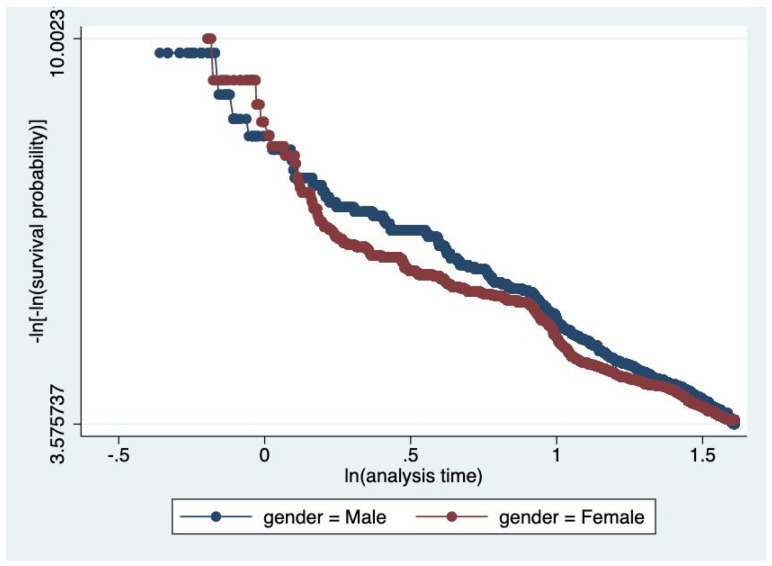
Complementary log–log (CLL) plot of survival by gender. Parallel curves suggest proportional hazards between male and female participants.

**Figure 5:**
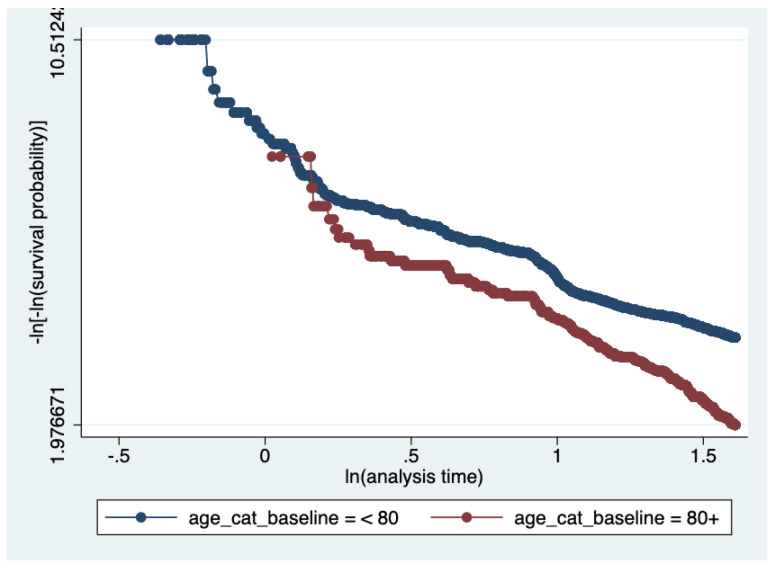
Complementary log–log (CLL) plot by age group. The rough parallel curves indicate proportional hazards between participants aged <80 and those aged ≥80 at baseline.

**Figure 6:**
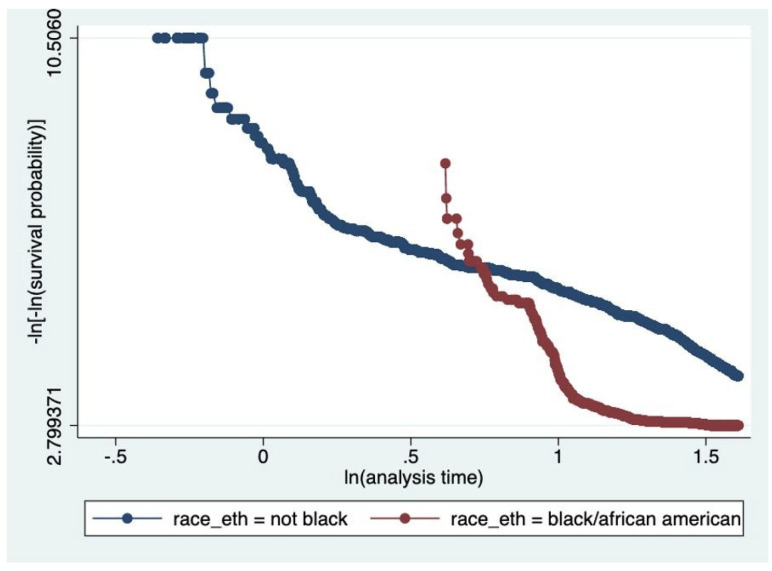
Complementary log–log (CLL) plot by race/ethnicity. The divergence in slopes suggests potential non-proportional hazards between Black/African American and non-Black participants.

Kaplan–Meier survival curves (Figure 1, Figure 2 and Figure 3) were plotted to examine the relationships between each of the primary covariates (gender, age at entry, and race) and time-to-death. Corresponding complementary log–log (CLL) plots (Figure 4, Figure 5 and Figure 6) were used to verify the proportional hazards assumption of a constant hazard ratio between different categories of the covariates over time.

Since AQI at study entry (>50 vs. ≤50) was the main point of interest, an unadjusted model was constructed using only AQI to predict time-to-event. The unadjusted hazard ratio indicated that patients with an AQI > 50 have a hazard of death that is 3.52 times higher than patients with an AQI of less than 50 (95% CI: 2.96–4.19, *p* < 0.05).

Similarly, five other unadjusted models were constructed, in which only either gender, age, race, region, or year of entry was used to predict time-to-event. The results of the unadjusted models are presented on the left panel of Table 2.

To identify potential confounders in the relationship between air quality and survival time, five bivariate models were constructed with two covariates each, AQI and either gender, age, race, region, or year of entry (Table 3).

As shown in Table 3, the AQI coefficient remained nearly the same in all five bivariate models, so it was concluded that none of the five covariates was distorting the effect of AQI on survival. Additionally, older age, Black/African American race, and later year of entry into the study (after 2016) were significantly associated with an increased hazard of death (*p* < 0.05), while gender was not significantly associated. However, gender was retained in the final model along with the other covariates because of its typically assumed association with death. There was a statistically significant reduction in hazard of death (*p* < 0.05) in most regions compared to the Mountain Region. Hazard of death increased with every year of entry from 2016 until 2019 and declined in the time period 2020–2022.

### 3.2. Time-Independent Analysis

The adjusted results of the time-independent multivariable Cox Regression Model are shown on the right panel of Table 2. Males and females had no difference in hazard of death. Patients older than 80 years had a 6.73 times higher hazard of death (95%CI: 5.47, 8.28; *p* < 0.05) than patients younger than 80 years, and Black/African American patients had a 4.27 times higher hazard of death (95% CI: 3.47, 5.26; *p* < 0.05) than non-Black/African American patients. In comparison to the Mountain region (reference category) of the United States, the East North Central, Pacific, South Atlantic, New England, and East South Central regions had significantly lower hazards of death (*p* < 0.05), while the West North Central and Middle Atlantic regions had significantly higher hazards of death (*p* < 0.05).

Earlier year of entry into the study was significantly associated with lower survival (*p* < 0.05). Compared to study entry in 2016, study entry in 2017, 2018, 2019, and 2020–2022 all had significantly higher hazards of death (*p* < 0.05). The hazard ratio increased from 2016 to 2017, 2017 to 2018, and 2018 to 2019. The hazard declined from 2019 to 2020–2022.

Finally, the hazard of death in the group with AQI > 50 was 4.02 times higher than the hazard of death in the group with AQI ≤ 50 (95% CI: 3.36, 4.82; *p* < 0.05).

## 4. Discussion

### 4.1. Time-Independent Analysis

Our analysis found that a higher Air Quality Index was statistically significantly associated with a higher hazard of mortality. Black/African American race, as well as older age, were statistically significantly associated with increased mortality. Earlier year of entry into the study was associated with lower survival, and regional effects played a role in survival as well. The relationship between year of study entry and mortality displayed a clear pattern. From 2016 to 2019, the hazard of death increased with year of entry. However, between 2019 and the period 2020–2022, the risk of death with year of entry appeared to decline. This stark decrease could be potentially attributed to the COVID-19 pandemic and lockdown, which resulted in a decline in outdoor air pollution. A 2020 World Air Quality Report claimed that “human-related emissions from industry and transport fell during lockdowns” and that air quality improved globally from 2019 to 2020 [18]. The hazard ratios when comparing the years 2017, 2018, and 2020–2022 to the reference year 2016 were between 2 and 3. However, the hazard ratio comparing 2019 to 2016 was 35.64. While we cannot definitively attribute this outlier to a single cause, several potential contributing factors exist. These include documented air quality disturbances in 2019 due to regional wildfire events, which disproportionately affected vulnerable populations in certain geographic areas [19]. Additionally, data irregularities—including small subgroup sample sizes or variations in reporting practices—likely have contributed to the observed effect. Excess mortality due to the COVID-19 spike, caused a lack of shelter-in-place protocols in the early stages of the pandemic, could contribute to the mortality spike seen in 2019.

### 4.2. Strengths

A key strength of this study is the use of a novel dataset that links electronic health record (EHR) data with real-world air quality exposures—an integration that remains rare in the existing literature. This linkage enables more granular assessments of environmental impacts on health. Future research would benefit from the creation and open sharing of similar integrated datasets to facilitate broader, population-level analyses across diverse settings.

To our knowledge, this is the first environmental epidemiology study using the American Family Cohort data. This study is longitudinal in nature, which allowed us to make robust conclusions regarding the effects of air quality on mortality, thereby laying a foundation for future causal association studies. A key strength of this study is that it analyzes data on underserved populations, including persons from racial and ethnic minorities. The AFC dataset covers more than 800 primary care practices across 47 states, with 40% of AFC patients residing in rural areas. AFC Data also includes a wide payer mix, with patients on private insurance plans, Medicaid, and Medicare, increasing the representation of vulnerable populations and the generalizability of the sample to the overall United States population. This study lays the groundwork for future studies, as the survival analysis methodologies from this study can be applied to other “time-to-event” analyses, such as determining the time to development of certain health outcomes, like various respiratory and cardiovascular diseases.

### 4.3. Limitations

This is an epidemiological study that utilizes EHR data that have already been accrued. Thus, while the data have the inherent strength of being real-world data, they are associated with some limitations.

We acknowledge that using AQI at baseline may not reflect longitudinal exposure. Future studies should seek to incorporate time-varying AQI measures to better capture chronic exposure and its temporal dynamics. Incorporating temporally resolved exposure data would be powerful in enhancing causal inference.

An additional limitation is the method by which the “race” variable was encoded. Our decision to dichotomize race as Black/African American vs. other was intentional, as we were specifically interested in examining disparities affecting Black/AA patients, who experience disproportionately higher rates of adverse health outcomes. Additionally, due to small sample sizes in several subgroups and data limitations, race was dichotomized. We recognize that doing so may mask important disparities and aim to incorporate more granular race/ethnicity categories in future analyses.

Another limitation was the fact that the models could not fully account for extreme or acute events such as forest fires. Such events are associated with specific dates and locations, and there does not currently exist a consolidated longitudinal dataset spanning the entire United States with these events demarcated by location and date.

A third limitation was the way in which the COVID-19 pandemic and its effects on survival were handled. Because most of the data were collected between the years of 2020 and 2022, it was not possible to account for COVID-19 directly by using a binary variable corresponding to whether the visit date was during the period of the pandemic (this variable was determined to be collinear during the statistical analysis and was therefore not included in the final model). We acknowledge that the COVID-19 pandemic may have introduced substantial confounding through excess mortality and altered healthcare access. Our current model does not account for the competing risks of COVID-related death, and future iterations will explore competing risk models to better isolate the effect of air quality.

The models could not fully account for extreme or acute events, such as forest fires, in the analysis. Such events are associated with a specific time and location, and there is no consolidated longitudinal dataset spanning the entire United States with these events demarcated by location and date. Incorporating these events into the analysis would add robustness to the model.

Finally, the lack of precision of the death data in this dataset is a potential limitation. Death data were not confirmed from the National Death Index and were reported by health-care providers or family members. We relied on death dates reported by families or providers, which may be subject to recall or reporting bias. Although this likely results in non-differential misclassification, linkage with the National Death Index or vital records is essential to ensure accuracy in future work.

### 4.4. Public Health Significance

Our study has significant policy implications for public health. A patient’s environment is not only an exposure but also a social determinant of health. As such, it is important for clinicians to understand their patients’ environmental conditions in order to provide the best possible care. Based on the results of this study, clinicians caring for older adults and Black/African American patients should use real-time air quality maps such as “Purple Air: Real Time Air Quality Map” to routinely assess the air quality in their patients’ environments [20]. Our findings support targeted policy interventions such as enforcing stricter air quality regulations in vulnerable regions, alert systems within healthcare settings for high-AQI days, and infrastructure investment in urban green spaces to mitigate pollution exposure.

Our findings align with the prior research demonstrating a link between long-term exposure to air pollution and increased all-cause mortality. Prior studies, including large-scale analyses of Medicare beneficiaries and multi-regional cohorts, have reported elevated risks of mortality associated with higher concentrations of PM2.5, nitrogen dioxide, and ozone. Di. et al., 2017, found that each 10 μg/m^3^ increase in annual PM2.5 was associated with a significant increase in the risk of mortality in older adults, even at concentrations below what is currently acceptable by EPA standards [21]. Wu et al., 2020, found that communities with historically higher air pollution burdens experienced disproportionate mortality during the COVID-19 pandemic, which further emphasizes the effects of environmental exposures as a determinant of health outcomes, especially in vulnerable groups [22].

In contrast to these prior studies, our study leverages the richness of longitudinal EHR data linked with AQI scores to enable the dynamic capture of exposure and outcome information across a diverse, real-world population. Our study is unique in that it uses pre-collected EHR data, providing a scalable framework for future studies, rather than relying on cohort recruitment which is inherently biased. Using EHR data, which is vast and detailed, can lay the foundation for future environmental surveillance and the development of interventions to improve public health.

From a translational perspective, the findings from our study have practical implications from both a clinical care and health policy perspective. Integrating environmental data into EHR systems could enable automated risk stratification tools that flag patients with chronic disease who reside in high-AQI areas. Healthcare systems could then use this information to tailor preventative care. For example, patients with COPD could be sent reminders on high AQI days, suggesting that they remain indoors and utilize an air purifier. Other patients residing in chronically high AQI zones could be referred to a cardiopulmonary specialist earlier than suggested by screening guidelines. At a policy level, the results of the study indicate a strong potential benefit to geographically targeted interventions, such as maintaining stricter EPA regulation enforcement in zip codes with chronically high pollution burden or creating an alert system to alert residents about instances of poor air quality in real time.

## 5. Conclusions

Our study shows that poor air quality is associated with a higher hazard of mortality. While all patients were adversely impacted by air pollution, we highlight that this phenomenon was particularly pronounced for Black/African American patients. Pollution accelerated mortality for older adults as well. The underlying etiology of these findings is beyond the scope of this study. Prospective studies are needed to understand the causal mechanisms underlying our findings.

Currently, we are in the process of incorporating the Neighborhood Atlas’ Neighborhood Deprivation Index data to examine how socioeconomic index and location play a role in the relationship between air quality and mortality.

We feel that our methodology could be extended to the analysis of the effects of poor air quality on the time to development of specific health outcomes, such as asthma, heart disease, and chronic obstructive pulmonary disease (COPD), as well as gender-specific health outcomes such as gynecological cancers. We hope to use a similar analysis methodology for these outcomes. The findings of our study can even potentially be extrapolated to a predictive model that can determine the risk of mortality based on air quality and a variety of demographic factors.

## Figures and Tables

**Table 1:** Patient demographics.

Covariate	Total *N* = 39,596 (100%)
**Gender**	
Female	56.00%
Male	44.00%
**Age**	
80+ years	7.04%
<80 years	92.96%
**Race**	
Black/African American	7.62%
Other	92.38%
**Region**	
East North Central	5.48%
West South Central	7.56%
West North Central	30.62%
Pacific	15.74%
South Atlantic	8.57%
Middle Atlantic	2.22%
New England	16.16%
East South Central	4.60%
Mountain	9.04%
**Year of Entry**	
2016	9.94%
2017	28.35%
2018	25.83%
2019	11.20%
2020–2022	24.67%

**Table 2:** Unadjusted and adjusted results of time-independent Cox Regression Models of time-to-death (* *p* < 0.05).

	Unadjusted	Adjusted
	HR 95% CI	HR 95% CI
**Covariate**		
**Air Quality**		
AQI > 50	3.52 * (2.96, 4.19)	4.02 * (3.36, 4.82)
AQI <= 50	1.00	1.00
**Gender**		
Female	1.11 (0.93, 1.32)	0.93 (0.78, 1.11)
Male	1.00	1.00
**Age**		
80 + years	4.69 * (3.82, 5.74)	6.73 * (5.47, 8.28)
<80 years	1.00	1.00
**Race**		
Black/African American	3.93 * (3.26, 4.23)	4.27 * (3.47, 5.26)
Other	1.00	1.00
**Region**		
East North Central	0.23 * (0.09, 0.55)	0.39 * (0.16, 0.96)
West South Central	1.15 (0.72, 1.84)	1.03 (0.64, 1.67)
West North Central	2.37 * (1.60, 3.51)	2.93 * (1.97, 4.36)
Pacific	0.23 * (0.12, 0.42)	0.29 * (0.15, 0.54)
South Atlantic	0.21 * (0.11, 0.43)	0.24 * (0.12, 0.49)
Middle Atlantic	1.21 (0.61, 2.39)	2.09 * (1.05, 4.16)
New England	0.76 (0.49, 1.18)	0.53 * (0.34, 0.82)
East South Central	0.14 * (0.05, 0.40)	0.24 * (0.08, 0.70)
Mountain	1.00	1.00
**Year of Entry**		
2016	1.00	
2017	2.19 * (1.58, 3.02)	3.33 * (2.38, 4.66)
2018	2.91 * (2.05, 4.14)	4.62 * (3.22, 6.63)
2019	10.90 * (7.41, 16.05)	35.64 * (23.68, 53.63)
2020–2022	1.90 * (0.96, 3.76)	4.24 * (2.10, 8.53)

**Table 3:** Two variable adjusted time-independent Cox Regression Models of time-to-death (* *p* < 0.05).

	Model A	Model B	Model C	Model D	Model E
Covariate	HR 95% CI	HR 95% CI	HR 95% CI	HR 95% CI	HR 95% CI
**Air Quality**					
AQI > 50	3.52 *	3.67 *	3.27 *	3.65 *	3.70 *
	(2.96, 4.19)	(3.08, 4.36)	(2.75, 3.89)	(3.06, 4.35)	(3.11, 4.40)
AQI <= 50	1.00	1.00	1.00	1.00	1.00
**Gender**					
Female	1.10				
	(0.92, 1.31)				
Male	1.00				
**Age**					
80+ years		4.99 *			
		(4.08, 6.11)			
<80 years		1.00			
**Race**					
Black/African American			3.34 *		
			(2.94, 4.26)		
Other			1.00		
**Region**					
East North Central				0.30 *	
				(0.13, 0.74)	
West South Central				1.31	
				(0.82, 2.10)	
West North Central				2.63 *	
				(1.77, 3.90)	
Pacific				0.28 *	
				(0.15, 0.52)	
South Atlantic				0.21 *	
				(0.10, 0.42)	
Middle Atlantic				1.50	
				(0.76, 2.96)	
New England				0.64	
				(0.41, 1.00)	
East South Central				0.21 *	
				(0.07, 0.61)	
Mountain				1.00	
**Year of Entry**					
2016					1.00
2017					2.31 *
					(1.67, 3.19)
2018					2.95 *
					(2.08, 4.19)
2019					12.40 *
					(8.42, 18.27)
2020–2022					2.39 *
					(1.21, 4.73)

## Data Availability

The data supporting the findings of this study are available for access at https://americanfamilycohort.org/ (accessed on 1 January 2023). Access may be subject to institutional review and data use agreements, as outlined on the website.
